# Motivational interviewing for prevention in Swiss family medicine: Opportunities and challenges

**DOI:** 10.1016/j.pmedr.2023.102351

**Published:** 2023-07-29

**Authors:** Maria Caiata-Zufferey, Carlo De Pietro

**Affiliations:** University of Applied Sciences and Arts of Southern Switzerland (SUPSI), Department of Business Economics, Health and Social Care (DEASS), Stabile Piazzetta, Via Violino 11, CH-6928 Manno, Switzerland

**Keywords:** Motivational interviewing, Implementation, Primary prevention, Family doctors, Medicalisation, Acceptability, Cost-effectiveness, Switzerland, Qualitative study

## Abstract

Health promotion and primary prevention are a priority in a healthcare system characterised by a prevalence of chronic conditions. In this context, motivational interviewing (MI) as provided by family doctors (FDs) seems promising: influential health professionals motivate patients to adopt healthy lifestyles in a patient-centred style that promotes a balanced, horizontal doctor-patient relationship. Based on these assumptions, a pilot project called Girasole was implemented in Switzerland between 2016 and 2018 to train and support 19 FDs in implementing MI in their practices. This paper presents the analysis of implementation of the intervention with the aim of exploring the doctors’ experiences with MI through a qualitative research design. Data derive from focus groups and interviews with the participants, and from the observation of collective training sessions and follow-up meetings. A thematic analysis was conducted using the software Atlas.ti. Results show that there is great diversity in how FDs implement MI. FDs can be classified in four groups – convinced, interested, critical, and resistant – based on their adherence to the principles underlying the MI approach. This taxonomy highlights opportunities and challenges for family medicine: MI offers flexible tools and new ways of interacting with patients to meet the challenges of non-communicable and chronic diseases; at the same time, the issues associated with the medicalisation of human everyday problems, physicians’ status loss, and low cost-effectiveness should not be underestimated. Any further attempt to promote MI among FDs should take into account their individual attitudes and should establish tailored approaches and training methods.

## Highlights

• This study examines the experience of family doctors with motivational interviewing.
• Family doctors have different views on the meaning of motivational interviewing.
• Implementation varies depending on the features of doctors, patients, and situations.
• Motivational interviewing supports family doctors in their usual preventive activity.
• Family doctors’ motivational interviewing poses economic and professional challenges.

## 1 Introduction

This article presents the results of a qualitative study conducted with 19 family doctors (FDs) who participated in Girasole, a pilot project implemented in Southern Switzerland between 2016 and 2018 to promote motivational interviewing (MI) in family medicine. Participants were trained in MI and invited to use it in their practice to encourage physical activity among patients. The objective of this paper is to present the analysis of the implementation of the intervention, specifically to provide insights into the experiences of FDs’ when integrating MI in their daily practice, and to discuss the potential benefits and challenges of this approach in family medicine.

Due to ageing populations and chronic illnesses, primary prevention that promotes healthy behaviours is increasingly necessary (Hajat and Stein, 2018). Primary care plays an important role in this context by assisting patients in identifying their health needs and priorities, and navigating the healthcare system effectively (Wallace et al., 2015). However, the literature shows that FDs do not systematically address healthy lifestyles with their patients, and when they do, discussions tend to be succinct (Bloy et al., 2016). According to many scholars, FDs lack tools and resources of energy, time, knowledge, and skills to engage in primary prevention (Geense et al., 2013, Rubio-Valera et al., 2014, Hall et al., 2022). Greater investment in the deliberate implementation of health promotion activities is therefore needed (Anstiss, 2009).

MI has drawn significant attention and debate in family medicine (Thepwongsa et al., 2017, Boom et al., 2022). Described as a “collaborative, person-centered form of guiding to elicit and strengthen personal motivation for change” (Miller & Rollnick, 2009, 137), MI is a communication approach that enhances patients’ autonomy and fosters a partnership dynamic between physicians and patients, encouraging information and expertise sharing for a healthier lifestyle (Anstiss, 2009). Though appearing as a smooth conversation, MI requires extensive practice and continuous learning (Miller & Rollnick, 2009). Core communication skills such as open-ended questioning, summarizing, and reinforcing change talk are essential to this approach, as well as behaviour change strategies like setting the scene, assessing importance and confidence, exploring options, and setting goals and a plan (Anstiss, 2009). MI provided by FDs seems promising in terms of efficacy and acceptability: lifestyle/health challenges are addressed by influential, well-known health professionals through a patient-centred approach that promotes a balanced doctor-patient relationship.

MI in primary care has been studied and promoted in many countries (McKenzie et al., 2018, Fatusin et al., 2021, Boom et al., 2022), but a systematic evaluation has proven difficult due to the heterogeneity of the interventions (VanBuskirk & Wetherell, 2014). Generally, there is consensus on the appropriateness of MI in primary care (Rubak et al., 2009, Boom et al., 2022), though debate remains concerning the training of physicians (Dragomir et al., 2019), long-term efficacy on patient behaviour (Thepwongsa et al., 2017, VanBuskirk and Wetherell, 2014, Rubak et al., 2009), and which health professionals are best suited to provide these services (VanBuskirk & Wetherell, 2014). FDs’ involvement in particular has been criticised as cost-ineffective (VanBuskirk & Wetherell, 2014), and little data exist on their experience with MI or on difficulties in implementing it in their daily practice (Thepwongsa et al., 2017, Boom et al., 2022).

## 2 Girasole and its approach

The Girasole pilot project was conceived and promoted by the Health Promotion and Assessment Service (HPAS), a cantonal division responsible for non-communicable disease prevention, health promotion, and health evaluation in Canton Ticino. This Italian-speaking region in Switzerland is characterized by a low rate of physically active individuals and a high frequency of FDs’ consultation (Petrini and Roth, 2014, Stanga, 2014). This made Ticino an ideal setting for implementing a pilot project focused on promoting physical activity through MI in primary care. A total of 19 FDs from across Ticino were invited to participate based on their previous experience or interest in health promotion, along with their personal connections with the HPAS. Funding was granted by the Swiss Federal Office of Public Health.

Developed between 2014 and 2016, Girasole combined two programmes previously implemented in French- and German-speaking Switzerland: Paprica, a training course developed by the University Medical Policlinic of Lausanne, aimed at enabling FDs to integrate patient counselling on physical activity into their daily practice (Koutaissoff et al., 2012); and Health Coaching, a programme of the Swiss College of Primary Care Medicine for behavioural counselling and health promotion in primary care (Neuner-Jehle et al., 2013). From Paprica, Girasole adopted the training concept and material on physical activity, while Health Coaching provided the general methodology of MI. Associated materials were developed for use across stages, including a directory of physical activities in Ticino and worksheets for tracking goals, means, and measures of behavioural changes over time. Girasole’s communicative approach was characterised by openness, empathy, and support of patient autonomy. FDs were invited to act as “coaches,” helping patients define their goals, means, and pace of behavioural change.

The project started in September 2016 with a two-day training session led by a FD specialized in psychosomatic medicine and skilled in MI, utilizing simulated consultations with actors. Following the training, FDs provided positive feedback through a questionnaire assessing its organization, content and relevance. The intervention phase spanned from October 2016 to March 2018. Over the course of 18 months, FDs were encouraged to integrate MI into their practice. They were asked to recruit adults aged 40–75 - a particularly sedentary population in Ticino (Petrini and Roth, 2014, Stanga, 2014) - who were willing to adopt a more active lifestyle. During the intervention phase, the HPAS offered post-training support through three meetings led by the same trainer for discussing challenges, sharing strategies, and fostering a learning-by-doing approach.

## 3 Methods

### 3.1 Theoretical and methodological framework

To explore physicians’ experience with MI, we conducted a qualitative thematic study following Braun and Clarke (2006). This approach was chosen for its ability to offer a comprehensive and detailed analysis of complex data. Considering MI as a social innovation, we adopted Dearing’s theory of innovation diffusion (2009) as a theoretical framework. This theory suggests that five components are key to understanding the diffusion of innovation: the innovation, the adopter, the social system, the individual adoption process, and the diffusion system. We focused on the dimensions of innovation, adopter, and individual adoption process to systematically explore the physicians’ perceived characteristics of MI, their readiness to use MI, and their implementation of MI into daily practice. Guided by this framework, we developed semi-structured guidelines for qualitative data collection through focus groups and interviews.

This study did not fall within the scope of Art. 2 and Art. 3 of Swiss law on human research and did not require ethics approval. However, all participants provided oral informed consent. The study was also conducted in accordance with the Declaration of Helsinki (World Medical Association, 2013).

### 3.2 Sample

The sample was composed of the totality of the 19 FDs selected to participate in Girasole. The sample represents 5.5% of the physicians in family medicine in Ticino at the time of the intervention in 2017 (FMH, 2022). As a result, the sample is not representative of the average situation of family medicine in Ticino or Switzerland, as young people, women, and doctors who share their practice with one or more colleagues are over-represented (Senn et al., 2016). The characteristics of the participants are summarised in Table 1.

**Table 1:** Characteristics of the participant family doctors.

Gender	13 women 6 men
Age	17 physicians = 36–55 y.o. 2 physicians > 56 y.o.
Race/ethnicity	White/European
Geographic area	8 physicians working in an urban area 7 physicians practicing in a semi-urban area 4 physicians practicing in a rural area
Kind of medical practice	3 physicians working in solo practice 9 physicians sharing their practice with a colleague 7 physicians working in group practices

### 3.3 Data collection

Data were collected twice: at the beginning of the intervention phase (Fall 2016) to explore participants’ attitudes, expectations, and previous involvement with health promotion in primary care; and at the end of it (Spring 2018) to evaluate participants’ experience with Girasole. Based on the participants’ availability and geographical proximity, we conducted five mini-focus groups and three individual interviews at the beginning of the project (with all 19 physicians) and four mini-focus groups and four individual interviews at the end (with 16 physicians involved; two had left the project after some weeks, while a third was unable to meet). Mini focus groups (up to 4 people) were privileged to harness group dynamics while gaining in-depth insights in participants’ experiences (Krueger & Casey, 2014). Individual interviews were conducted in specific cases due to participant preferences and organizational constraints.

Discussions were based on semi-structured guidelines with open-ended questions (e.g., “Think about the patients to whom you proposed MI this week. How did you decide to offer them MI?”), but participants were encouraged to address any aspects they considered important. The focus groups were facilitated by both authors, whereas the interviews were carried out by the first author. All sessions were conducted in person. Data were audio recorded, transcribed, and anonymised. Additionally, both authors participated as observers in the two-day training session and the three follow-up meetings, taking extensive notes to enrich the narrative dataset. Focus groups, interviews, and follow-up meetings lasted 60–120 min.

### 3.4 Data analysis

Data were analysed thematically following Braun and Clarke’s approach (2006), which intends to identify, analyse and report patterns within data through a series of iterative phases: familiarizing with the data by re-reading the transcripts, coding the data, identifying themes, reviewing themes, creating relationships between themes, and writing the report. The first author conducted the coding process inductively, assigning codes to manifest and latent content. Regular meetings were held between the two authors throughout the whole analysis process to discuss coding and interpretation. Disagreements were resolved through discussion and by continuously referencing to the data. At the end of the analysis, a consensual general codebook was developed and utilized to recode all the transcripts with the software Atlas.ti. This process enabled the assessment of the relevance and comprehensiveness of the results, the verification of the data saturation, and the identification of relevant quotations. Results were presented to participants during a final session in Spring 2019, obtaining respondent validation.

## 4 Results

We have analysed the implementation of MI by FDs using three dimensions of the innovation diffusion theory (Dearing, 2009): the innovation, the adopter, and the individual adoption process. In the first paragraph (innovation) we explore participants’ perception of the Girasole approach. In the second paragraph (adopter) we focus on the FDs’ readiness to engage in MI. The third paragraph (individual adoption process) highlights how FDs’ concretely incorporate MI in their daily practice. A fourth and last paragraph provides an overview of the findings and proposes a taxonomy of the physicians based on their degree of adherence to the principles of the Girasole approach. Quotes from participants are provided in Table 2 and identified by number. To protect physicians’ privacy, the quotations are anonymous.

**Table 2:** Exemplary quotes from the participant family doctors.

Themes	Quote #	Supporting quotes
Innovation	1	*This project is interesting for me because it allows me to better develop an aspect that was already part of my work before.*
	2	*I realise that, basically, the way I was doing prevention – although with a certain sensitivity – I was perhaps a bit directive in my attitude. On the other hand, being able to experiment with a more participative dimension, perhaps by turning a little more around the problem to give patients the possibility of expressing their doubts*… *it’s stimulating.*
	3	*The great strength [of Girasole] is that each of us [participating physicians] has used it differently. It’s a strength, you can adapt it to your personality.*
	4	*This morning I wanted the patient to tell me that she could have gone for a walk after lunch. According to motivational interviewing, I had to say: ‘Do you ever think that you could go for a walk?’ And the patient looked at me: ‘Boh.’ And then I said: ‘For example, after dinner!’ And I thought: ‘What an idiot!’ Because according to motivational interviewing, this is something you shouldn’t do. But in the end, you want to follow the rules of motivational interviewing so much that you end up in absurd dead ends when the patient would just like you to say: ‘After dinner? Great idea!’*
	5	*It is this structure that irritates me or makes me sceptical. It takes away the spontaneity of the relationship. If things are too structured*… *in the end, the patient realises whether it’s me talking or it’s a preconceived programme that goes through my mouth.*
	6	*We doctors are supposed to deal with pathology, not with ‘healthology.’*
	7	*Patients come here to have a doctor in front of them, someone who makes a diagnosis and prescribes treatments. If I ask: ‘What do you want to do to solve your situation?’, I am told: ‘It is up to you to tell me: you are the doctor!’*
	8	*It is a project that enhances the role of the general practitioner.*
	9	*It’s a different approach. Patients no longer hear the doctor’s moralising speech: ‘Do this, do that,’ but they are more in the centre, they are the ones who have to do something. It's good that they set the objective and that you help them achieve it. It is stimulating and even makes the consultation enjoyable.*
	10	*It can also be an instrument that makes the work more interesting. It gives a certain impetus to the profession.*
	11	*This is the strength of this programme: you can work on a theme chosen by the patient, and automatically you will also address the others [themes]. Because they are interrelated.*
	12	*If patients agree, but then fail to do what they have decided, you can always work on the reasons for failure.*
	13	*There is a double risk: time management, but also content management. In other words, there is uncertainty: how to manage the time and how to handle what comes out. Because we are not psychologists.*
	14	*It’s a problem that will never be solved. You work 10–12 h a day, the shops close at 6.30p.m., you eat poorly because if you don’t go out at 6p.m. you can’t do the shopping.*
Adopter	15	*Health promotion is part of the ADN of the family doctor.*
	16	*Our work is something that goes far beyond diagnosis and therapy.*
	17	*I believe in prevention like this: you are 50 years old, and from now on, it is essential that for the next 10 years – unless you have specific symptoms – you have a colonoscopy. This is the only prevention we can do.*
	18	*I always tell them [patients] that they can do much more than I can do. Because I can go and put a stent in when you have a heart attack, but then your lifestyle, you have to work on it continuously.*
	19	*It’s better to work a little bit on the lifestyle, because if I send him to the cardiologist, he comes out with five prescribed drugs.*
	20	*You have to be realistic: we all have a hard life. If you take away my 10 cigarettes a day, what’s left? Smoke, eat, go to the bar, have a drink, get off work and have a beer*…*.*… *It’s a way of pampering the soul.*
	21	*Most people work 10 h a day, have a family (*…*) I also say to myself: ‘I have to go dancing, I have to go running, I have to take care of myself’ (*…*) Yes, and then? Let’s be serious. Life doesn’t work like that.*
Individual adoption-process	22	*Even if the patient refuses to participate, it allows a seed to be planted that could blossom later.*
	23	*I choose the one who is predisposed. And predisposed means someone who is already somewhat motivated.*
	24	*It must be a patient I feel comfortable with. Because we are human beings, there are some people we like to invest more time with, but there are others who are less desirable.*
	25	*It depends on my agenda. If I am already stressed because the afternoon is full, it’s not good because I wouldn’t even manage properly. So I don’t do it.*
	26	*I offer it to the person who comes for that [lifestyle risk factor]. For example, someone who comes because in the last few years he has gained 23 kg. I say: ‘He came for that, I can offer it.’ However, if the person comes for sinusitis, even if he is obese, no. Unless they ask themselves.*
	27	*I made myself a list. I saw, for example, that Mrs X was coming and I planned a longer appointment. I made it longer, yes, I made it longer. But it's essential, otherwise you won’t make it.*
	28	*I proposed it like that, in the consultation, because the opportunity arose.*
	29	*I find it difficult to tell a patient to come back to the practice, but if you have goals and ask the patient to come back after three months to evaluate them, it’s okay to do that. But forcing her to do so was embarrassing.*
	30	*For example, someone came, did the blood tests, and I said: ‘Okay, so next time we’ll discuss the blood tests and then we’ll also talk about that [Girasole], if you agree.’*
	31	*It doesn’t take that much to impress, it’s a question of attitude.*
	32	*I asked him: ‘Would you like to do something for your health?’ And the patient started crying. And then it’s quarters and quarters of hours and quarters of hours going away, you know. [This approach is] Beautiful and impossible.*
	33	*The concept is correct, it is intelligent, and it makes sense, but it should not be followed mechanically. It is a partially helpful instrument if you manage to go your own way. After all, the patient is following me, not a programme.*

### 4.1 Innovation

Most participants rated the Girasole approach positively in terms of *process of use* and *effect*. They considered it *handy*, as it was accessible (it could be applied despite the short training), structuring (it improved the current health promotion activities) (#1–2), and flexible (it was adaptable to physicians’ and patients’ needs) (#3). Some participants thought it was *constraining* (protocol and tools were sometimes considered artificial and intrusive) (#4–5). Only a few found it *incompatible* with their daily practice due to workload constraints and a professional identity focused solely on the treatment of illness (#6–7).

Most participants recognized the benefits of the Girasole approach in terms of *transforming* their professional activity, rather than exclusively focusing on patients’ behavioural change: the Girasole approach provided legitimacy to their ongoing health promotion practices (#8), enhanced the gratification of consultations (making them more pleasant and interesting) (#9–10), and fostered generative discussions where new actions could be explored with the patient (#11–12). Some participants perceived the approach as *risky* (introducing uncertainty into the consultation) (#13), while a minority viewed it as *utopian* (believing that MI was bound to fail due to the predominance of socio-economic factors on lifestyle) (#14).

### 4.2 Adopter

Two key aspects influenced physicians’ adoption of the Girasole approach: their view of *health promotion* in medical practice and their approach to lifestyle *risk factors*. Regarding health promotion, all FDs considered it central to their role (#15–16). However, there were varying positions among them: some were willing to prioritize their *patients’ lifestyles*, while others focused more on disease and *secondary prevention* (#17).

Risk factors were likewise understood differently. One group was determined to *address risk factors*, emphasizing the role of lifestyle in determining health outcomes (#18–19). A larger group tended to interpret and sometimes *justify unfavourable health behaviours*, arguing that they were meaningful to the patients (#20). A final group *accepted risk factors* as resulting from social conditions beyond the control of patients and physicians (#21). These various attitudes influenced both physicians’ readiness to participate in the project and their ongoing commitment to it.

### 4.3 Individual adoption process

Physicians adapted the Girasole approach to their professional styles and habits, developing a variety of methods for the *identification*, *recruitment*, *follow-up organisation*, and *coaching* of patients. Regarding identification, a few physicians made an *extended selection*: they offered MI systematically by integrating it “routinely” into all consultations, as they believed that every patient could benefit from MI at any given moment (#22). Other physicians made a *targeted selection*, based on the characteristics of the patient (i.e., their receptiveness or friendliness) (#23–24) or on context (i.e., time) (#25–26).

Regarding recruitment, some physicians *scheduled* specific MI consultations (#27). Others would *improvise*, at the risk of not having enough time (#28). Still others briefly introduced MI during regular consultations, but *rescheduled* appointments ad hoc.

To follow up, some physicians arranged *dedicated consultations*, despite feeling embarrassed about summoning patients and subsequently charging them for reasons that were both not strictly clinical and not solicited by the patients themselves (#29). Others chose to *incorporate* follow-up within regular consultations (#30), while some *left the initiative* to the patients.

As for coaching, we observed three forms of conducting MI. A few participants used “*expert” coaching*, feeling confident and managing adequately time and MI tools (#31). Most practitioners adopted an “*amateur” coaching* style, appreciating the approach, but feeling they were lacking training and experience; they used MI selectively with comfortable patients, yet struggled with time and content control during consultations (#32). Others provided a *“personalised” coaching* approach, seeing MI as a general model but taking liberties in adapting style and materials; they emphasized the physician’s authority and charisma over specific techniques (#33).

### 4.4 Taxonomy of physicians

According to their accounts, physicians displayed diverse perspectives and practices regarding MI. A closer examination of the data reveals that the key factor underlying this variation is physicians’ *adherence to the Girasole approach* and its four core principles: importance of health promotion to FDs; legitimacy of FDs’ intervention in patients’ lifestyles; appropriateness of the motivational approach; and mastery of motivational techniques and tools. Assessing the level of adherence to these principles allows for the classification of participants into four ideal types: resistant, critical, interested, and convinced.

Two physicians can be characterized as *resistant*: they showed reluctance in addressing health behaviours and patients’ lifestyles, instead favouring curative measures or secondary prevention. They considered the Girasole approach utopian and incompatible with their professional identity and abandoned the project after a few weeks. Two other participants, designated *critical*, showed curiosity in new patient interactions but maintained an asymmetric relationship and did not fully embrace active listening, open questions, and patient empowerment. They believed the Girasole approach constrained their naturalness in patient interactions and adopted a personalised coaching style. The largest group, consisting of 11 *interested* physicians, were willing to intervene in patients’ health behaviours and lifestyles using a motivational approach. However, they faced challenges in applying its tools and techniques due to limited expertise, concerns about patient selection, and time constraints. They felt uncertain about the impact of MI on consultations and adopted an “amateur” coaching style. While intrigued by the approach, they lacked confidence in mastering it, resulting in inconsistent and selective application. In contrast, a final group of four *convinced* physicians fully embraced the Girasole approach. They found it valuable and transformative, and employed MI with suitable expertise. The taxonomy is summarised in Table 3.

**Table 3:** The taxonomy of family doctors based on their adherence to the Girasole approach.

	**Convinced**	**Interested**	**Critical**	**Resistant**
	*“It doesn’t take that much to impress, it’s a question of attitude”*	*“Beautiful and impossible”*	*“After all, the patient is following me, not a programme”*	*“We doctors are supposed to deal with pathology, not with ‘healthology’”*
**Adherence to the founding principles of the Girasole approach**				
Health Promotion	Yes	Yes	Yes	Yes
Patient lifestyle intervention	Yes	Yes	Yes	No
Motivational approach	Yes	Yes	No	No
Motivational techniques and tools	Yes	No	No	No
**Perception of the Girasole approach**	Handy Transformative	Risky	Constraining	Incompatible Utopian
**Type of MI performed**	Expert	Amateur	Personalised	

## 5 Discussion

Girasole aimed to incorporate MI into primary care to promote physical activity among patients at risk. The qualitative analysis confirmed physicians’ interest and adoption of MI in their practice, indicating its feasibility. Previous research supports the importance of addressing patients’ health behaviour for FDs and its potential impact on job satisfaction (Boom et al., 2022). However, implementing MI in primary care faces barriers such as time constraints, patient-provider relationship, and patient characteristics (e.g., social background, level of understanding), as highlighted in the literature (Östlund et al., 2015, Reno et al., 2018, Jager et al., 2020, Boom et al., 2022). Our study confirms these findings, demonstrating that diverse attitudes and approaches exist within a group of FDs selected for their interest in health promotion. Therefore, achieving systematic use of MI for health promotion may prove challenging, even among motivated and interested physicians.

Beyond feasibility, our results also show that MI has several potential advantages in family medicine. First, MI provided structure, tools, and legitimacy to ongoing preventive activities. This suggests that more systematic work in health promotion and prevention could help FDs better define their professional identity and social role in contrast to specialist physicians. Second, according to the participants, patients appreciated MI and felt that prevention had a place in consultations. However, this study provides no evidence on the impact of MI on patients’ behaviour, which remains difficult to measure outside controlled clinical/pharmaceutical environments.

Some aspects of MI are puzzling. Medicalisation, for instance, has always been a thorn in the side of preventive interventions. Medicalisation stands for “the expansion of medical authority into the domains of everyday existence” (Metzl & Herzig, 2007, 697). In Girasole, physicians proposed health promotion activities to people who presented no symptoms of disease and who did not necessarily ask for prevention advice. Some FDs expressed their discomfort on this point, which raises ethical questions about adopting MI. These are all the more crucial when the physician must emphasise the risks connected to a patient’s behaviour. The patient becomes labelled “at risk,” which can lead to anxiety, fear, diminished self-esteem, malaise, and stigmatisation (Gérvas et al., 2008). A second problematic point concerns the status and legitimacy that FDs can gain from carrying out systematic health promotion interventions. In this study we observed that the lifestyle recommendations were largely common sense (e.g., avoiding a sedentary lifestyle), as were the methods to achieve these goals (e.g., having a dog to walk). This highlights two weaknesses in FDs’ practice of MI: the commonplace of the issues discussed, and the borrowing of techniques from other professional groups (e.g., psychologists or social workers). These weaknesses may question the legitimacy of FDs as healthcare providers. A third issue pertains to the cost-efficiency of FDs in conducting MI. Nearly all participants identified time as a challenge for MI. With a shortage of physicians in primary care, there are arguments in favour of prioritizing curative activities (Gérvas et al., 2008). Involving other and less costly healthcare professionals, such as midwives or nurses (Weingarten and Matalon, 2010), could present a potentially cost-effective alternative. Furthermore, there is a question of whether the promotion of health should be primarily addressed by the community and public policy, focusing on aspects like ensuring the availability of affordable and healthy food. Of course, this is not an either/or debate: community action and public policy can coexist with MI conducted by FDs. However, the efficacy of the latter remains uncertain, as confirmed by the participants who expressed resistance.

This study has some limitations. Since participants were selected based on their interest in health promotion, diversity was limited, even though we were able to partially address this point by interviewing the two doctors who left the project after training. Additionally, FDs were given a questionnaire to provide feedback on the training, but the skills acquired during training were not measured. Finally, data relied on physicians’ narratives, but direct observation of MI in the clinical setting and exploration of its impact on patients were not feasible.

Still, this study offers a comprehensive understanding of physicians’ implementation of MI. The taxonomy provides a general framework for understanding FDs’ positions towards MI and their ways of implementing it. This may be used for statistical studies of the profiles of FDs in relation to health promotion and MI, and for developing tailored training to the different groups. Any further attempt to implement programmes like Girasole should recognise the existence of different individual attitudes and define customised approaches and training.

## 6 Conclusion

This analysis of the Girasole pilot project provides new inputs for discussions on the feasibility, efficacy, and acceptability of MI in family medicine. Findings show that MI is a viable activity that offers flexible tools and new ways of interacting with patients to meet the challenges of non-communicable and chronic diseases. The implementation of this approach and the training programmes should consider the diversity of physicians’ attitudes towards health promotion and intervention in patients’ lifestyle, and their familiarity with MI and its techniques and tools. At the same time, the issues of medicalisation, physicians’ status loss, and low cost-effectiveness should not be underestimated.

**Sources of funding**

This work was supported by the Swiss Federal Office of Public Health (grant no. 16.022197/704.0001-790/2). The sponsor did not have any involvement in the design and realisation of the study or in the writing of the paper.

## Declaration of Competing Interest

The authors declare that they have no known competing financial interests or personal relationships that could have appeared to influence the work reported in this paper.

## Data Availability

The data that has been used is confidential.

## References

[b0005] Anstiss T. (2009). Motivational interviewing in primary care. J. Clin. Psychol. Med. Settings.

[b0010] Bloy G., Moussard Philippon L., Rigal L. (2016). Les médecins généralistes et le conseil en activité physique: des évidences aux contingences de la consultation. Sante Publique.

[b0015] Boom S.M., Oberink R., Zonneveld A.J., van Dijk N., Visser M.R. (2022). Implementation of motivational interviewing in the general practice setting: a qualitative study. BMC Prim Care.

[b0020] Braun V., Clarke V. (2006). Using Thematic Analysis in Psychology. Qual. Res. Psychol..

[b0025] Dearing J.W. (2009). Applying diffusion of innovation theory to intervention development. Res. Soc. Work. Pract..

[b0030] Dragomir A.I., Julien C.A., Bacon S.L., Boucher V.G., Lavoie K.L. (2019). Training physicians in behavioural change counseling: A systematic review. Patient Educ. Couns..

[b0035] Fatusin B.B., Yakubu K., Engmann S.T., Fatusin A.J., Oseni T.I.A., Opare-Lokko E.B.A., Ayisi-Boateng N.K., Ayodapo A.O. (2021). Motivational interviewing: Exploring its relevance for improving family medicine trainees' motivation in Sub-Saharan Africa. Nigerian J. Family Practice.

[b0040] Geense W.W., Van De Glind I.M., Visscher T.L., Van Achterberg T. (2013). Barriers, facilitators and attitudes influencing health promotion activities in general practice: an explorative pilot study. BMC Fam. Pract..

[b0045] Gérvas J., Starfield B., Heath I. (2008). Is clinical prevention better than cure?. Lancet.

[b0050] Hajat C., Stein E. (2018). The global burden of multiple chronic conditions: A narrative review. Prev. Med. Rep..

[b0055] Hall L.H., Thorneloe R., Rodriguez-Lopez R., Grice A., Thorat M.A., Bradbury K., Wadnerkar Kamble M., Okoli G.N., Powell D., Beeken R.J. (2022). Delivering brief physical activity interventions in primary care: a systematic review. Br. J. Gen. Pract..

[b0060] Jager M., den Boeft A., Leij-Halfwerk S., van Der Sande R., van den Muijsenbergh M. (2020). Cultural competency in dietetic diabetes care - A qualitative study of the dietician’s perspective. Health Expect..

[b0065] Koutaissoff D., Jeannin A., Dubois-Arber F. (2012).

[b0070] Krueger R.A., Casey M.A. (2014).

[b0075] McKenzie K.J., Pierce D., Gunn J.M. (2018). Guiding patients through complexity: Motivational interviewing for patients with multimorbidity. Austral. J. General Practice.

[b0080] Metzl J.M., Herzig R.M. (2007). Medicalisation in the 21st century: Introduction. Lancet.

[b0085] Miller W.R., Rollnick S. (2009). Ten things that motivational interviewing is not. Behav. Cogn. Psychother..

[b0090] Neuner-Jehle S., Schmid M., Grüninger U. (2013). The “Health Coaching” programme: a new patient-centred and visually supported approach for health behaviour change in primary care. BMC Fam. Pract..

[b0095] Östlund A.S., Wadensten B., Kristofferzon M.L., Häggström E. (2015). Motivational interviewing: Experiences of primary care nurses trained in the method. Nurse Educ Practice.

[b0100] Petrini, L. & Roth, S. (2014). *Rapport de base sur la santé pour le canton du Tessin. Exploitations standardisées des données de l’Enquête suisse sur la santé 2012 et d’autres bases de données*. Obsan Dossier 38. Neuchâtel : Observatoire suisse de la santé.

[b0105] Reno J.E., O’Leary S., Garrett K., Pyrzanowski J., Lockhart S., Campagna E., Barnard J., Dempsey A.F. (2018). Improving provider communication about HPV vaccines for vaccine-hesitant parents through the use of motivational interviewing. J. Health Commun..

[b0110] Rubak S., Sandbæk A., Lauritzen T., Borch-Johnsen K., Christensen B. (2009). General practitioners trained in motivational interviewing can positively affect the attitude to behaviour change in people with type 2 diabetes: One year follow-up of an RCT, ADDITION Denmark. Scand. J. Prim. Health Care.

[b0115] Rubio-Valera M., Pons-Vigués M., Martínez-Andrés M., Moreno-Peral P., Berenguera A., Fernández A., Harper D.M. (2014). Barriers and facilitators for the implementation of primary prevention and health promotion activities in primary care: a synthesis through meta-ethnography. PLoS One.

[b0120] Senn, N., Ebert, S.T. & Cohidon, C. (2016). *La médecine de famille en Suisse. Analyse et perspectives sur la base des indicateurs du programme SPAM (Swiss Primary Care Active Monitoring). Obsan Dossier 55.* Neuchâtel : Observatoire suisse de la santé.

[b0125] Stanga M. (2014). “Come va la vita ?” L’indagine tematica sulla salute 2012: alcuni risultati per la Svizzera e la Svizzera italiana. Dati – Statistiche e società.

[b0130] Thepwongsa I., Muthukumar R., Kessomboon P. (2017). Motivational interviewing by general practitioners for Type 2 diabetes patients: a systematic review. Family Pract.

[b0135] VanBuskirk K.A., Wetherell J.L. (2014). Motivational interviewing with primary care populations: a systematic review and meta-analysis. J. Behav. Med..

[b0140] Wallace E., Salisbury C., Guthrie B., Lewis C., Fahey T., Smith S.M. (2015). Managing patients with multimorbidity in primary care. BMJ.

[b0145] Weingarten M., Matalon A. (2010). The ethics of basing community prevention in general practice. J. Med. Ethics.

[b0150] World Medical Association (2013). World Medical Association Declaration of Helsinki: Ethical principles for medical research involving human subjects. J. Am. Med. Assoc..

